# Ophthalmic nurses in vision centres in Bangladesh

**Published:** 2020-12-31

**Authors:** Mohammed Gowth Amanullah, Golam Mostafa, Sathi Raha Bhodendranath

**Affiliations:** 1 Senior Faculty: Lions Aravind Institute of Community Ophthalmology (LAICO), Aravind Eye Care System, Madurai, Tamil Nadu, India.; 2 Director, Professor and Line Director: National Eye Care, National Institute of Ophthalmology, Sher – Bangla Nagar, Dhaka, Bangladesh.; 3 Vision technician: Community Vision Centre – Upazilla Health complex, Lohagara Bangladesh.

**Bangladesh has adopted a structured approach to train general nurses as vision technicians, in order to address the shortage of trained eye care professionals in rural areas.**

**Figure:**
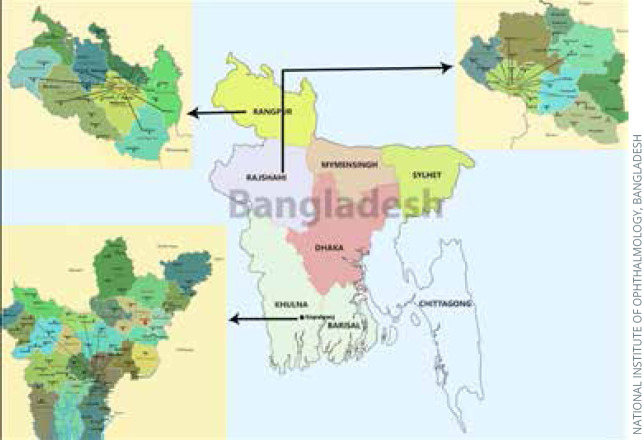
Map of Bangladesh showing the locations of various vision centres. **BANGLADESH**

In Bangladesh, eye care services are provided by the government, private hospitals, and local and international non-governmental organisations. However, access and coverage are still inadequate, as most ophthalmologists are located in urban areas, whereas 63 per cent of the country's population resides in rural areas. The current cataract surgical rate is 1,950 while the rate needed to address the backlog as well as new (incident) cases is estimated at 6,000. The Cataract Surgical Rate (CSR) is defined as the number of cataract operations done per million population per year.

Bangladesh requires a robust, scalable, and innovative solution to meet the need for cataract surgery and other eye services in the country. The health ministry therefore adopted a model developed by the Aravind Eye Care System. Under the model, trained ophthalmic personnel at the institute's telemedicine enabled vision centres (VCs) will offer eye care services at the primary level.

## Government's role

The government decided to integrate primary eye care in the portfolio of primary health care services by setting up community vision centres. After making the necessary budgetary allocation, the government appointed a senior team consisting of the Director: National Institute of Ophthalmology, senior ophthalmologists, and heads of teaching hospitals in Bangladesh, to visit Aravind Eye Hospital in India. The team studied Aravind's Vision Centre model in detail and developed a strategic road map for implementing a similar model in Bangladesh.

## The roadmap

The team decided to set up the first 20 community vision centres around a government tertiary eye hospital which would provide teleconsultation services, provide surgery, advanced investigations, and treatment to patients referred by the community vision centres. The team also decided that the community vision centers should be established in Upazila health complexes. Upazila refers to the third administrative level in Bangladesh and each of these covers a population of 100,000 - 200,000 people. The plan is to have a vision centre in each of the 500 Upazilas eventually.

Bangladesh has sufficient general nurses trained, available, and are working in various Government health care facilities. Hence, the team decided to select and train general nurses as vision technicians to work at the vision centres. The selection criteria, although strict, was designed to maximise staff retention. Nurses had to:

- live near the Upazila health complex
- be willing to learn new skills and switch to a career in ophthalmology
- be aged between 25 and 40 years.

## Training

The nurses who were selected had to undergo two months of intense training at the National Institute for Ophthalmology in Bangladesh. This covered:

- basic eye care
- anatomy and functions of the eye
- handling of basic ophthalmic equipment
- visual acuity measurement
- subjective refraction
- identification of common eye conditions.

After training the nurses were assessed for their knowledge and skills. This was followed by a 45-day intense training programme at Aravind Eye Hospital, the mentoring institute and its vision centres. This program provided hands-on training in the following areas:

### Ophthalmic skills

These included visual acuity measurement, subjective refraction, retinoscopy, slit lamp examination, tonometry, identifying common eye conditions, fundus photography, ocular pharmacology, and treatment options for different eye conditions.

**Table 1:** Performance up to December 2019

Base hospital	Months of operation (since)	No of CVCs	Population covered	New outpatient visits	New outpatient to population coverage	Review outpatient visits	Total outpatient	Average per day
SFMEHTI, Gopalgonj	16 (Aug 2018)	20	3,853,000	1,03,686	2.7%	5,702	109,388	18
MC, Rajshahi	9 (Apr 2019)	15	3,443,475	36,176	1.1%	1,991	38,167	15
MC, Rangpur	9 (Apr 2019)	15	3,981,071	33,307	0.7%	1,143	34,450	13
**Total**		**50**	**11,277,546**	**173,169**	**1.5%**	**8,979**	**1,82,005**	**21**
**Now 7% of Bangladesh's population has permanent access to eye care**								

### Information technology

This focused on the use of electronic medical records, and teleconsultation between patients and ophthalmologists at the base hospital.

### Patient engagement

Counselling to enhance compliance with treatment.

### Management

Managing supplies, equipment maintenance and generate reports.

The mentoring team at Aravind assessed nurses' new skills. On their return to Bangladesh, the newly qualified vision technicians then returned to the National Institute of Ophthalmology to continue learning and practising their skills until the first vision centres were launched.

## Launch of community vision centres in Bangladesh

A team from Aravind Eye Hospitals, comprising the programme manager, vision technicians and an IT expert, worked closely with the team in Bangladesh, to set up the first 12 community vision centres. This helped to iron out first issues and ensured the smooth functioning of the community vision centres.

To build a cohesive working relationship between the vision technicians and the ophthalmologists at the tertiary hospital, both groups were invited to attend a workshop at the tertiary eye hospital, where the speakers emphasised the importance of the community vision centres and the vital role they play in extending effective eye care services to the rural population of Bangladesh.

## Services delivered at community vision centre

- Carrying out comprehensive eye examination supported by teleconsultation with ophthalmologists.
- The vision technicians offer the services like dispensing spectacles and medicines as per e-prescription from ophthalmologists.
- Those requiring cataract surgery or advanced care are referred to the hospital.

Currently, there are 50 community vision centres in Bangladesh. Twenty are linked to a tertiary eye hospital (Sheikh Fazilatunnessa Mujib Eye Hospital and Training Institute), and 15 each are linked to two medical colleges (Rajshahi Medical College and Rangpur Medical College).

