# Supplementary material for: Ophthalmic nurses in vision centres in Bangladesh
Source: Community Eye Health. 2020 Dec 31;33(110):S5–6. (PMC8115705)
Supplement: Related texts

# Brief report: From the field

**Published:** 2020

**Sister Sathiraha Bhodendranath** is a general nurse who underwent intensive training to become a vision technician at one of Bangladesh's 50 community vision centres.

“I was working as a staff nurse in Upazila health complex in Lohagara, Khulna when I applied to train as a vision technician. I have always wanted to help people to regain their eyesight, so I was very motivated! The fact that vision technicians do not work night shifts was also very attractive.

The interviews were very competitive, so I was delighted to be one of the first group of 20 nurses selected to attend the initial training at the National Institute for Ophthalmology (NIO).

The shift from general nursing to ophthalmology was very challenging in the beginning. The two months of training at NIO boosted my confidence, and subsequent practical training at Aravind, with the continuous feedback they offered, really helped me to master the required skills.

I now see 20 to 25 patients per day, on my own; it is very satisfying work! The number of outpatients that come to us is increasing steadily, and the positive feedback from our patients is a motivating factor. The highlight of my career as a vision technician was when I demonstrated the vision centre model at the Digital Bangladesh Mela in 2020 with my colleague Mrs Smirti. We won an award at this Mela. I am working now as a vision technician in the community vision centre at Lohagara.”
